# Impact on plant productivity under low-fertility sandy soil in arid environment by revitalization of lentil roots

**DOI:** 10.3389/fpls.2022.937073

**Published:** 2022-08-04

**Authors:** Mohamed A. Abd El-hady, Yasser M. Abd-Elkrem, Mohamed O. A. Rady, Elsayed Mansour, Khaled A. El-Tarabily, Synan F. AbuQamar, Mohamed E. El-temsah

**Affiliations:** ^1^Agronomy Department, Faculty of Agriculture, Ain Shams University, Cairo, Egypt; ^2^Agronomy Department, Faculty of Agriculture, Fayoum University, Fayoum, Egypt; ^3^Agronomy Department, Faculty of Agriculture, Zagazig University, Zagazig, Egypt; ^4^Department of Biology, College of Science, United Arab Emirates University, Al Ain, United Arab Emirates; ^5^Khalifa Center for Genetic Engineering and Biotechnology, United Arab Emirates University, Al Ain, United Arab Emirates; ^6^Harry Butler Institute, Murdoch University, Murdoch, WA, Australia

**Keywords:** carbohydrates, nitrogen uptake, nodulation, phosphorus uptake, proteins, root activator, yield traits

## Abstract

Lentil is one of the essential legume crops, which provides protein for humans and animals. This legume can improve soil fertility through nitrogen fixation, which is imperative in low-fertility soils. The growth and productivity of lentil could be enhanced through improving nutrition and root revitalization. Therefore, the objective of this study was to assess the impact of root activator (RA) and phosphorus (P) application on morphological, physiological, agronomic, and quality traits of lentil under newly reclaimed low-fertility sandy soil in an arid environment. The RA was applied at four levels of 0 (RA0-untreated control), 1.25 (RA1), 2.5 (RA2), and 3.75 (RA3) l ha^–1^. RA contained 9% potassium humate, 1,600 ppm indole butyric acid, 200 ppm gibberellic acid, and 200 ppm naphthalene acetic acid. The recommended rate of phosphorus (P) fertilization in the newly reclaimed low-fertility sandy soil (75 kg P_2_O_5_ ha^–1^) was applied, and its amount was increased and decreased by 25 kg P_2_O_5_ ha^–1^ vs. non-added control. Thus, P rates were applied at four rates 0 (P0; control), 50 (P1), 75 (P2), and 100 (P3) kg phosphorus pentoxide (P_2_O_5_) ha^–1^. Our results revealed that treated lentil plants with the high levels of both treatments (RA3 and P3) exhibited superiority in root measurements (root length, total number of nodules plant^−1^, number of active nodules plant^−1^, dry weights of active nodules, and total root), nitrogenase activity, chlorophyll *a* and *b*, carotenoids, yield traits, and seed proteins and carbohydrates. However, the recommended P level (75 kg P_2_O_5_ ha^–1^, P2) under the high level of RA (3.75 l ha^–1^, RA3) displayed non-significant differences in yield traits (plant height, 1,000-seed weight, seed yield ha^–1^) and quality traits (protein and carbohydrate) with the high P level (100 kg P_2_O_5_ ha^–1^, P3). Accordingly, its recommended economically and environmentally to use this coapplication of RA3 and P3 in low-fertility soil for better lentil growth, and seed yield and quality.

## Introduction

Lentil (*Lens culinaris* Medic.) is an edible legume grown broadly and cultivated for its nutritious seeds (Ganesan and Xu, 2017). Its global cultivated area is almost 5.01 million hectares that produce around 6.53 million tons (FAOSTAT, 2022). It is one of the cheapest sources of vegetable protein and provides a plentiful amount of minerals, fibers, and fundamental amino acids (Khazaei et al., 2019). Additionally, its straw is an alternative unconventional feedstuff for livestock (Jarpa-Parra, 2018). It is a palatable and nourishing feed for animals that contributes significantly to cope with continuously increasing forage demands (Mudgal et al., 2018). Moreover, it fixes atmospheric nitrogen (N_2_) and reduces the utilization of synthetic nitrogenous fertilizers, accordingly diminishes greenhouse gas emissions (Schmidtke et al., 2004; Khazaei et al., 2019). Symbiotic fixation of atmospheric N_2_ increases the content of mineral nitrogen (N) in the soil, which remains after lentil harvesting (Hossain et al., 2016). Accordingly, it considerably enriches soil fertility and improves its biological properties, particularly in newly reclaimed low-fertility soils (Gan et al., 2017; Liu et al., 2019; Desoky et al., 2021c).

Newly reclaimed sandy soils suffer from a deficiency of available mineral nutrients, and subsequently low-crop productivity (Mansour et al., 2017; Omar et al., 2022). Hence, numerous endeavors are adopted to elevate legume productivity and quality under newly reclaimed soils (Dhaliwal et al., 2021; El-Mageed et al., 2021). This could be accomplished through improving nutrition approaches, which had a pivotal impact on plant growth and productivity (Mannan et al., 2022). Enhancing root architecture is imperative to ameliorate resource acquisition, water uptake, plant anchoring, and encourage the utilization of soil nutrients (Gahoonia and Nielsen, 2004; Mansour et al., 2021).

Potassium (K) humate is a highly effective plant growth bio-activator for stimulating root development and penetration, photosynthesis efficiency, plant growth, and tolerance against environmental stress (Pradip et al., 2017). Moreover, it contains soluble humic that boosts cell division, synthesis of proteins and nucleic acids, tissue regeneration, and movement of nutrients (Shah et al., 2018; Jindo et al., 2020). Furthermore, the plant growth regulators as butyric acid (BA), gibberellic acid (GA_3_), and naphthalene acetic acid (NAA) promote impacts on roots, particularly under environmental stresses (Egamberdieva et al., 2017; Sabagh et al., 2021; ElShamey et al., 2022). Growth regulators enhance root initiation, cell division, cell elongation, cell differentiation, vascular tissue, and apical dominance (Singh, 2010; Semida et al., 2021).

Phosphorus (P) is an essential macronutrient for legume development and growth. It plays a decisive role in invigorating biological activities, such as symbiotic N_2_ fixation by *Rhizobium*, prolific root hair formation, and nutrient uptake (Míguez-Montero et al., 2020). Furthermore, P application expands the number of root nodules, regulates their growth, enhances nitrogenase activity, and accordingly improves the capacity of N_2_ fixation (Gahoonia and Nielsen, 2004). Moreover, it is involved in various fundamental functions, such as cell division, nucleus formation, energy transfer, sugar transformation, nutrient movement, photosynthesis, seed formation, protein synthesis, and crop ripening (Swailam et al., 2021). Accordingly, its application ameliorates root development, biological activities, physiological capabilities, and metabolic functions, which lead to significant enhancement of legume productivity, particularly under newly reclaimed soil conditions (Singh and Singh, 2016).

Different studies have considered the influence of mineral elements on lentil plants. However, further studies are required to explore the responses of lentils to combinations of important elements, such as K humate, indole butyric acid (IBA), GA_3_, and NAA as root activator (RA), as well as P fertilization under newly reclaimed low-fertility sandy soil in arid environments. Therefore, the objective of the present study was to assess the impact of RA and P application at different levels of physiological, morphological, agronomic, and quality traits of lentil under newly reclaimed low-fertility sandy soil in arid environments.

## Materials and Methods

### Experimental site, soil, and climatic conditions

A field experiment was performed at the Higher Institute for Agricultural Cooperation Farm, Regwa region, Alexandria Desert Road, Beheira Governorate, Egypt (30°11′12.0′′N, 30°34′32.7′′E during the 2018/2019 and 2019/2020 growing seasons. The physical and chemical properties of the experimental soil were determined before sowing and presented in Supplementary Table 1. The experimental soil was sandy throughout the profile (92.1% sand, 1.6% silt, and 6.3% clay), with a pH of 7.5, and the electrical conductivity was 0.8 dS m^–1^.

The available nutrients were 30.72 mg N, 5.12 mg P, and 24.32 mg K kg^–1^ of the soil. Accordingly, this soil suffers from a considerable deficiency of available mineral nutrients, particularly NPK compared to fertile soils. The monthly minimum and maximum temperatures and total rainfall for the two growing seasons were obtained from a station close to the experimental site (Supplementary Table 2).

### Experimental design and agronomic practices

The RA used in this study contained 9% K humate, 1,600 ppm IBA, 200 ppm GA_3_, and 200 ppm NAA. The RA was applied at four levels, 0 (RA0; untreated control), 1.25 (RA1), 2.5 (RA2), and 3.75 (RA3) l ha^–1^ after 20 days after sowing (DAS) as fertigation. The recommended rate in the newly reclaimed low-fertility sandy soil (75 kg P_2_O_5_ ha^–1^) was applied, and this amount was increased and decreased by 25 kg P_2_O_5_ ha^–1^ vs. non-added control. The P was also applied at four levels 0 (P0), 50 (P1), 75 (P2), and 100 (P3) kg phosphorus pentoxide (P_2_O_5_) ha^–1^.

The P source used was calcium superphosphate that contained 15.5% P_2_O_5_, 19.5% calcium, and 11.5% sulfur at pH 2.0. P fertilizer was added to the soil during its preparation for cultivation. N fertilizer was applied at a rate of 45 kg N ha^–1^ as ammonium nitrate (33% N) as fertigation in three equal doses at sowing (0), 15 and 30 DAS. K fertilizer was applied at a rate of 100 kg K ha^–1^ as potassium sulfate (K_2_SO_4_, 48% K_2_O) at soil preparation.

The experimental design was conducted using a split-plot arrangement in a randomized complete block design with three replicates. RA was the first factor, assigned to the main plots, and the rate of P was the second factor allocated to the sub-plots. Each plot comprised of five rows, 4 m long and 0.60 m wide, with two seeds were sown per hill, which were spaced 0.15 m apart. Each replicate included 16 plots, i.e., four RA rates × four P levels, 16 treatments in total, as shown in Tables 1–6. The tested genotype in this experiment was Giza-51, a commercial cultivar in Egypt, and was obtained from the Agricultural Research Center, Giza, Egypt. The sowing was applied during the first week of November in both seasons according to the optimal period for growing lentil in the region.

**TABLE 1:** Impact of root activator (RA) and phosphorus (P) level on root length (cm), number of nodules plant^–1^, dry weight of active nodules (mg plant^–1^), total root dry weight (mg plant^–1^), and nitrogenase enzyme activity (μmol C_2_H_4_ g^–1^ nodule dry weight h^–1^) in lentil plants.

Factor		Root length	Total number of nodules plant^–1^	Number of active nodules plant^–1^	Dry weight of active nodules	Total root dry weight	Nitrogenase enzyme activity
**RA**							
RA0 (control)		23.12 ± 1.17^d^	12.85 ± 0.75^d^	11.48 ± 0.72^d^	61.71 ± 3.89^d^	1022 ± 29.42^d^	7.78 ± 0.18^d^
RA1 (1.25 l ha^–1^)		28.60 ± 1.61 ^c^	15.33 ± 1.13 ^c^	13.62 ± 1.00 ^c^	73.45 ± 2.73^c^	1181 ± 27.81^c^	8.76 ± 0.23 ^c^
RA2 (2.50 l ha^–1^)		36.10 ± 1.01 ^b^	21.21 ± 1.15 ^b^	19.12 ± 1.28 ^b^	102.21 ± 2.99^b^	1314 ± 23.35^b^	11.13 ± 0.78 ^b^
RA3 (3.75 l ha^–1^)		43.57 ± 0.62 ^a^	29.38 ± 1.42 ^a^	26.75 ± 1.34 ^a^	142.97 ± 4.68^a^	1490 ± 30.19^a^	15.23 ± 0.72 ^a^
**P**							
P0 (control)		28.01 ± 2.52^d^	14.22 ± 1.54^d^	13.24 ± 1.33^d^	70.05 ± 3.89^d^	1175 ± 65.53^c^	8.40 ± 0.49^d^
P1 (50 kg P_2_O_5_ ha^–1^)		31.04 ± 2.78^c^	18.54 ± 1.99^c^	15.41 ± 1.85^c^	81.93 ± 4.87^c^	1224 ± 59.32^b,c^	10.38 ± 0.97 ^c^
P2 (75 kg P_2_O_5_ ha^–1^)		35.14 ± 2.22^b^	22.36 ± 2.00^b^	20.33 ± 1.98^b^	109.47 ± 3.91^b^	1271 ± 54.61^b^	11.80 ± 1.03^b^
P3 (100 kg P_2_O_5_ ha^–1^)		37.20 ± 1.84^a^	23.66 ± 2.15^a^	21.98 ± 1.96^a^	118.89 ± 4.65^a^	1337 ± 50.35^a^	12.32 ± 1.06^a^
**Interaction**							
RA0	P0	18.54 ± 0.08^o^	9.09 ± 0.05^n^	8.75 ± 0.18^c^	47.47 ± 1.62^c^	953.3 ± 10.17^i^	6.84 ± 0.11^k^
	P1	20.35 ± 0.09^n^	12.12 ± 0.07^c^	9.63 ± 0.20^k^	51.23 ± 1.11^kl^	983.0 ± 13.06^i^	7.77 ± 0.13^i,j^
	P2	25.33 ± 0.09^k^	14.77 ± 0.12^j^	13.26 ± 0.26^i^	70.94 ± 2.53^i^	1039 ± 15.63^h,i^	8.22 ± 0.12^g,h,i^
	P3	28.27 ± 0.18^j^	15.44 ± 0.16^i^	14.27 ± 0.14^h^	77.18 ± 1.76^h^	1114 ± 14.50^g,h^	8.29 ± 0.11^g,h^
RA1	P0	22.23 ± 0.10^m^	10.10 ± 0.06^m^	10.06 ± 0.21^j,k^	53.01 ± 1.14^jk^	1046 ± 13.71^h,i^	7.60 ± 0.12^j^
	P1	24.70 ± 0.11^c^	13.47 ± 0.08^k^	10.70 ± 0.23^j^	56.92 ± 1.23^j^	1180 ± 15.28^f,g^	8.63 ± 0.14^g^
	P2	31.87 ± 0.12^h^	18.47 ± 0.18^h^	15.90 ± 0.15^g^	86.34 ± 1.83^g^	1213 ± 12.02^e,f,g^	9.20 ± 0.10^f^
	P3	35.57 ± 0.15^f^	19.30 ± 0.20^g^	17.83 ± 0.17^f^	97.55 ± 1.96^f^	1283 ± 11.93^d,e,f^	9.60 ± 0.08^f^
RA2	P0	31.18 ± 0.21^i^	15.76 ± 0.06^i^	14.01 ± 0.10^h,i^	74.10 ± 1.53^h,i^	1203 ± 13.83^e,f,g^	8.03 ± 0.07^h,i,g^
	P1	35.03 ± 0.24^g^	19.70 ± 0.08^g^	16.10 ± 0.11^g^	85.68 ± 1.61^g^	1310 ± 10.17^c,d,e^	9.23 ± 0.08^f^
	P2	38.10 ± 0.15^e^	23.73 ± 0.15^e^	21.80 ± 0.15^d^	117.98 ± 1.31^d^	1341 ± 16.23^c,d^	12.83 ± 0.06^d^
	P3	40.10 ± 0.11^d^	25.63 ± 0.16^d^	24.57 ± 0.17^c^	131.06 ± 1.94 ^c^	1403 ± 11.12 ^b,c,d^	14.43 ± 0.14 ^c^
RA3	P0	40.10 ± 0.16^d^	21.94 ± 0.11^f^	20.16 ± 0.12^e^	105.64 ± 1.64^e^	1497 ± 18.63^a,b^	11.13 ± 0.08^e^
	P1	44.07 ± 0.17^c^	28.87 ± 0.14^c^	25.20 ± 0.15^c^	133.86 ± 1.81^c^	1423 ± 14.53^a,b,c^	15.90 ± 0.11^b^
	P2	44.87 ± 0.07^b^	32.46 ± 0.12^b^	30.37 ± 0.40^b^	162.61 ± 2.17 ^b^	1491 ± 13.02^a,b^	16.93 ± 0.14^a^
	P3	45.27 ± 0.08^a^	34.27 ± 0.21^a^	31.27 ± 0.18^a^	169.78 ± 1.01^a^	1548 ± 17.52^a^	16.97 ± 0.18^a^

**ANOVA**	**df**	***P-value***					

RA	3	<0.001	<0.001	<0.001	<0.001	0.001	<0.001
P	3	<0.001	<0.001	<0.001	<0.001	0.001	<0.001
RA × P	9	<0.001	<0.001	<0.001	<0.001	0.032	<0.001

Means followed by different letters under the same factor are significantly different according to Tukey’s HSD test (p ≤ 0.05).

**TABLE 2:** Impact of root activator (RA) and phosphorus (P) level on chlorophyll *a*, chlorophyll *b*, and carotenoids contents (mg 100 g^–1^ fresh weight) in lentil plants.

Factor			Chlorophyll *a*	Chlorophyll *b*	Carotenoids
**RA**					
RA0 (control)			0.52 ± 0.01^d^	0.25 ± 0.01^d^	0.31 ± 0.03^d^
RA1 (1.25 l ha^–1^)			0.61 ± 0.02^c^	0.28 ± 0.02^c^	0.38 ± 0.05^c^
RA2 (2.50 l ha^–1^)			0.82 ± 0.02^b^	0.44 ± 0.01^b^	0.83 ± 0.03^b^
RA3 (3.75 l ha^–1^)			0.91 ± 0.03^a^	0.53 ± 0.02^a^	1.16 ± 0.07^a^
**P**					
P0 (control)			0.60 ± 0.04^d^	0.31 ± 0.03^d^	0.48 ± 0.08^d^
P1 (50 kg P_2_O_5_ ha^–1^)			0.70 ± 0.05^c^	0.34 ± 0.04^c^	0.57 ± 0.10^c^
P2 (75 kg P_2_O_5_ ha^–1^)			0.76 ± 0.04^b^	0.39 ± 0.03^b^	0.73 ± 0.11^b^
P3 (100 kg P_2_O_5_ ha^–1^)			0.81 ± 0.04^a^	0.46 ± 0.03^a^	0.91 ± 0.11^a^
**Interaction**					
RA0		P0	0.43 ± 0.009^k^	0.19 ± 0.007^k^	0.18 ± 0.01^i^
		P1	0.50 ± 0.010^j,k^	0.21 ± 0.008^k^	0.21 ± 0.01^i^
		P2	0.57 ± 0.010^i^	0.26 ± 0.005^i,j^	0.36 ± 0.01^h^
		P3	0.58 ± 0.008^h,i^	0.32 ± 0.005^g,h^	0.50 ± 0.01^g^
RA1		P0	0.48 ± 0.010^k^	0.21 ± 0.008^k^	0.20 ± 0.01^i^
		P1	0.56 ± 0.012^i,j^	0.24 ± 0.009^j,k^	0.24 ± 0.01^i^
		P2	0.66 ± 0.011^g,h^	0.30 ± 0.006^h,i^	0.42 ± 0.02^g,h^
		P3	0.73 ± 0.010^f^	0.38 ± 0.006^f^	0.67 ± 0.02^f^
RA2		P0	0.71 ± 0.013^f,g^	0.37 ± 0.008^f,g^	0.69 ± 0.02^e,f^
		P1	0.81 ± 0.015^d,e^	0.42 ± 0.009^e,f^	0.78 ± 0.02^d,e^
		P2	0.86 ± 0.012^c,d^	0.46 ± 0.011^c,d,e^	0.87 ± 0.02^d^
		P3	0.90 ± 0.007^b,c^	0.50 ± 0.003^b,c^	0.97 ± 0.003^c^
RA3		P0	0.76 ± 0.012^e,f^	0.45 ± 0.006^d,e^	0.83 ± 0.06^d^
		P1	0.92 ± 0.015^b,c^	0.50 ± 0.007^b,c^	1.04 ± 0.08^c^
		P2	0.95 ± 0.008^b^	0.52 ± 0.005^b^	1.27 ± 0.03^b^
		P3	1.03 ± 0.037^a^	0.62 ± 0.033^a^	1.50 ± 0.05^a^

**ANOVA**	**df**		***P-value***		

RA	3		<0.001	<0.001	<0.001
P	3		<0.001	<0.001	<0.001
RA × P	9		0.008	0.053	<0.001

Means followed by different letters under the same factor are significantly different according to Tukey’s HSD test (p ≤ 0.05).

**TABLE 3:** Impact of root activator (RA) and phosphorus (P) levels on lentil seed yield and its attributes.

Factor		Plant height (cm)	Number of branches plants^–1^	Number of pods plant^–1^	1000 seed weight (g)	Seed yield (kg ha^–1^)	Biological yield (kg ha^–1^)
**RA**							
RA0 (control)		28.57 ± 0.88^d^	3.42 ± 0.14^c^	28.02 ± 1.24^d^	24.60 ± 0.41^d^	835 ± 39.5^d^	2318 ± 71.95^d^
RA1 (1.25 l ha^–1^)		34.67 ± 1.38^c^	4.02 ± 0.21^b^	38.69 ± 2.18^c^	28.13 ± 0.56^c^	1193 ± 63.27^c^	3721 ± 127.96^c^
RA2 (2.50 l ha^–1^)		42.96 ± 0.77^b^	4.24 ± 0.19^a^	45.09 ± 1.07^b^	30.57 ± 0.80^b^	1406 ± 65.85^b^	4146 ± 124.86^b^
RA3 (3.75 l ha^–1^)		49.01 ± 0.92^a^	4.21 ± 0.22^a^	46.58 ± 1.08^a^	31.75 ± 0.57^a^	1481 ± 71.03^a^	4276 ± 99.67^a^
**P**							
P0 (control)		34.35 ± 2.68^d^	3.24 ± 0.7^d^	32.78 ± 2.38^d^	25.86 ± 0.71^d^	952 ± 63.33^d^	3021 ± 208.51^d^
P1 (50 kg P_2_O_5_ ha^–1^)		38.43 ± 2.57^c^	3.49 ± 0.09^c^	38.26 ± 2.33^c^	28.49 ± 0.73^c^	1146 ± 60.87^c^	3589 ± 234.99^c^
P2 (75 kg P_2_O_5_ ha^–1^)		41.05 ± 2.13^b^	4.29 ± 0.09^b^	42.50 ± 2.18^b^	29.89 ± 1.06^b^	1361 ± 93.78^b^	3863 ± 241.24^b^
P3 (100 kg P_2_O_5_ ha^–1^)		41.38 ± 2.07^a^	4.87 ± 0.11^a^	44.83 ± 2.12^a^	30.80 ± 0.86^a^	1456 ± 86.01^a^	3989 ± 259.56^a^
**Interaction**							
RA0	P0	24.04 ± 0.11^j^	2.88 ± 0.004^j^	21.97 ± 0.33^m^	22.69 ± 0.17^h^	632 ± 2.59^m^	1925 ± 11.40^n^
	P1	27.96 ± 0.12^i^	3.03 ± 0.005^i^	26.75 ± 0.40^c^	24.65 ± 0.19^g^	824 ± 3.38^c^	2341 ± 13.86^m^
	P2	30.73 ± 0.19^h^	3.74 ± 0.004^e,f^	30.60 ± 0.24^j^	24.74 ± 0.12^g^	900 ± 7.98^k^	2498 ± 7.85^c^
	P3	31.54 ± 0.17^g^	4.03 ± 0.03^d^	32.75 ± 0.31^i^	26.34 ± 0.24^f^	985 ± 9.01^j^	2511 ± 13.17^c^
RA1	P0	27.96 ± 0.12^i^	3.20 ± 0.005^h^	28.53 ± 0.42^k^	25.22 ± 0.19^g^	903 ± 3.70^k^	2961 ± 14.53^k^
	P1	32.90 ± 0.15^f^	3.44 ± 0.005^g^	35.67 ± 0.53^h^	28.33 ± 0.21^e^	1128 ± 4.63^i^	3525 ± 12.88^i^
	P2	38.90 ± 0.25^e^	4.45 ± 0.005^c^	43.10 ± 0.34^e^	28.53 ± 0.18^e^	1262 ± 8.41^g^	4096 ± 12.87^g^
	P3	38.93 ± 0.18^e^	4.97 ± 0.04^b^	47.47 ± 0.46^c^	30.43 ± 0.14^c^	1479 ± 9.82^d^	4304 ± 10.08^e^
RA2	P0	38.73 ± 0.11^e^	3.49 ± 0.03^g^	39.38 ± 0.17^g^	26.33 ± 0.20^f^	1102 ± 6.13^i^	3459 ± 10.98^j^
	P1	43.03 ± 0.12^d^	3.84 ± 0.03^e^	45.27 ± .0.20^d^	30.27 ± 0.24^c^	1299 ± 7.23^f^	4193 ± 13.31^f^
	P2	44.93 ± 0.14^c^	4.46 ± 0.005^c^	46.77 ± 0.23^c^	32.73 ± 0.13^b^	1578 ± 6.76^c^	4403 ± 14.37^d^
	P3	45.13 ± 0.12^c^	5.18 ± 0.04^a^	48.93 ± 0.14^b^	32.93 ± 0.15^a,b^	1644 ± 5.50^b^	4527 ± 8.84^b^
RA3	P0	46.64 ± 0.29^b^	3.41 ± 0.05^g^	41.24 ± 0.25^f^	29.20 ± 0.31^d^	1173 ± 8.26^h^	3738 ± 7.03^h^
	P1	49.83 ± 0.31^a^	3.65 ± 0.06^f^	45.37 ± 0.28^d^	30.73 ± 0.27^c^	1333 ± 9.07^e^	4297 ± 9.78^e^
	P2	49.63 ± 0.12^a^	4.50 ± 0.08^c^	49.53 ± 0.28^a,b^	33.57 ± 0.16^a^	1703 ± 10.76^a^	4455 ± 11.42^c^
	P3	49.93 ± 0.18^a^	5.28 ± 0.02^a^	50.17 ± 0.18^a^	33.50 ± 0.15^a^	1714 ± 7.68^a^	4614 ± 10.03^a^

**ANOVA**	**df**	***P-value***					

RA	3	<0.001	<0.001	<0.001	<0.001	<0.001	<0.001
P	3	<0.001	<0.001	<0.001	<0.001	<0.001	<0.001
RA × P	9	<0.001	<0.001	<0.001	<0.001	<0.001	<0.001

Means followed by different letters under the same factor are significantly different according to Tukey’s HSD test (p ≤ 0.05).

**TABLE 4:** Impact of root activator (RA) and phosphorus (P) levels on protein and carbohydrate yields of lentil seeds.

Factor		Protein yield (kg ha^–1^)	Carbohydrate yield (kg ha^–1^)
**RA**			
RA0 (control)		143.87 ± 10.96^d^	414.96 ± 23.77^d^
RA1 (1.25 l ha^–1^)		230.47 ± 19.95^c^	680.20 ± 42.23^c^
RA2 (2.50 l ha^–1^)		341.80 ± 22.47^b^	770.83 ± 41.43^b^
RA3 (3.75 l ha^–1^)		379.52 ± 22.89^a^	797.83 ± 43.03^a^
**P**			
P0 (control)		186.87 ± 21.40^d^	478.92 ± 35.35^d^
P1 (50 kg P_2_O_5_ ha^–1^)		247.75 ± 25.50^c^	635.73 ± 40.91^c^
P2 (75 kg P_2_O_5_ ha^–1^)		311.27 ± 34.95^b^	750.91 ± 56.13^b^
P3 (100 kg P_2_O_5_ ha^–1^)		349.77 ± 30.94^a^	799.43 ± 51.72^a^
**Interaction**			
	P0	92.75 ± 1.25^c^	293.55 ± 1.82^c^
RA0	P1	133.30 ± 1.79^k^	405.64 ± 2.25^k^
	P2	157.34 ± 1.78^j^	454.52 ± 2.77^j^
	P3	192.11 ± 1.55^i^	506.13 ± 2.70^i^
	P0	147.22 ± 1.98^j^	465.96 ± 2.89^j^
RA1	P1	202.22 ± 2.12^i^	669.47 ± 3.16^f^
	P2	244.87 ± 2.32^h^	735.09 ± 3.35^e^
	P3	327.56 ± 1.97^e^	850.28 ± 4.82^d^
	P0	235.05 ± 1.70^h^	560.82 ± 5.03^h^
RA2	P1	311.44 ± 1.58^f^	734.82 ± 6.59^e^
	P2	395.24 ± 1.56^c^	880.19 ± 6.04^c^
	P3	425.46 ± 2.08^b^	907.48 ± 4.09^b^
	P0	272.47 ± 1.22^g^	595.35 ± 7.77^g^
RA3	P1	344.03 ± 1.59^d^	732.97 ± 9.57^e^
	P2	447.62 ± 1.99^a^	929.18 ± 5.07^a^
	P3	453.95 ± 1.52^a^	933.83 ± 9.91^a^

**ANOVA**	**df**	***P-value***	

RA	3	<0.001	<0.001
P	3	<0.001	<0.001
RA × P	9	<0.001	<0.001

Means followed by different letters under the same factor are significantly different according to Tukey’s HSD test (p ≤ 0.05).

**TABLE 5:** Impact of root activator (RA) and phosphorus (P) levels on N in seed (%), N in straw (%), total N uptake (kg ha*^–^*^1^), N recovery efficiency (NRE), and N use efficiency (NUE).

Factor		N in seeds	N in straw	Total N uptake	NRE	NUE
**RA**						
RA0 (control)		2.71 ± 0.086^d^	0.33 ± 0.009^d^	27.95 ± 1.94^d^	79.85 ± 5.55^d^	23.86 ± 1.12^d^
RA1 (1.25 l ha^–1^)	3.03 ± 0.095^c^	0.38 ± 0.011^c^	46.56 ± 3.78^c^	133.04 ± 10.80^c^	34.09 ± 1.80^c^	
RA2 (2.50 l ha^–1^)		3.85 ± 0.083^b^	0.46 ± 0.009^b^	67.29 ± 4.05^b^	192.26 ± 11.58^b^	40.16 ± 1.88^b^
RA3 (3.75 l ha^–1^)		4.07 ± 0.065^a^	0.47 ± 0.008^a^	74.02 ± 3.95^a^	211.48 ± 11.29^a^	42.30 ± 2.02^a^
**P**						
P0 (control)		3.02 ± 0.070^d^	0.37 ± 0.017^d^	37.81 ± 4.26^d^	108.04 ± 12.19^d^	27.21 ± 1.80^d^
P1 (50 kg P_2_O_5_ ha^–1^)		3.36 ± 0.095^c^	0.41 ± 0.020^c^	49.91 ± 5.20^c^	142.60 ± 14.86^c^	32.74 ± 1.73^c^
P2 (75 kg P_2_O_5_ ha^–1^)		3.53 ± 0.079^b^	0.42 ± 0.018^b^	60.51 ± 6.46^b^	172.88 ± 18.47^b^	38.88 ± 2.67^b^
P3 (100 kg P_2_O_5_ ha^–1^)		3.76 ± 0.068^a^	0.45 ± 0.017^a^	67.59 ± 6.02^a^	193.12 ± 17.20^a^	41.59 ± 2.45^a^
**Interaction**						
RA0	P0	2.35 ± 0.033^k^	0.30 ± 0.002^j^	18.75 ± 0.23^n^	53.58 ± 0.87^n^	18.05 ± 0.07^m^
	P1	2.59 ± 0.036^j^	0.32 ± 0.017^h,j^	26.14 ± 0.25^m^	74.70 ± 0.93^m^	23.53 ± 0.09^c^
	P2	2.80 ± 0.016^h,i^	0.34 ± 0.009^h^	30.54 ± 0.13^c^	87.25 ± 0.97^c^	25.72 ± 0.31^k^
	P3	3.12 ± 0.046^g^	0.37 ± 0.010^g^	36.36 ± 0.72^k^	103.89 ± 1.16^k^	28.15 ± 0.25^j^
	P0	2.61 ± 0.037^i,j^	0.33 ± 0.007^h,i^	30.32 ± 0.46^c^	86.62 ± 1.33^c^	25.79 ± 0.10^k^
RA1	P1	2.87 ± 0.040^h^	0.37 ± 0.008^g^	41.20 ± 0.63^j^	117.72 ± 1.80^j^	32.24 ± 0.13^i^
	P2	3.10 ± 0.029^g^	0.39 ± 0.003^f^	50.33 ± 0.27^h^	143.81 ± 0.79^h^	36.07 ± 0.24 ^g^
	P3	3.54 ± 0.053^e,f^	0.43 ± 0.004^e^	64.41 ± 0.69^f^	184.02 ± 1.12^f^	42.25 ± 0.65^d^
	P0	3.41 ± 0.032^f^	0.42 ± 0.006^e,f^	47.38 ± 0.34^i^	135.38 ± 0.99^i^	31.47 ± 0.17^i^
RA2	P1	3.84 ± 0.036^c,d^	0.46 ± 0.006^c,d^	63.03 ± 0.46^f^	180.08 ± 1.12^f^	37.11 ± 0.20^f^
	P2	4.01 ± 0.030^b,c^	0.47 ± 0.009^b^	76.61 ± 0.33^d^	218.88 ± 0.96^d^	45.09 ± 0.19^c^
	P3	4.14 ± 0.031^a,b^	0.49 ± 0.005^a,b^	82.15 ± 0.35^c^	234.72 ± 1.01^c^	46.97 ± 0.15^b^
	P0	3.72 ± 0.055 ^de^	0.44 ± 0.002^d,e^	54.80 ± 0.45^g^	156.58 ± 1.43^g^	33.51 ± 0.37^h^
RA3	P1	4.13 ± 0.061^a,b^	0.48 ± 0.003^b^	69.27 ± 0.77^e^	197.92 ± 1.07^e^	38.08 ± 0.43^e^
	P2	4.21 ± 0.023^a,b^	0.47 ± 0.010^b,c^	84.56 ± 0.55^b^	241.59 ± 1.58^b^	48.66 ± 0.36^a^
	P3	4.24 ± 0.026^a^	0.51 ± 0.016^a^	87.45 ± 0.48^a^	249.85 ± 1.05^a^	48.98 ± 0.21^a^

**ANOVA**	**df**	***P-value***				

RA	3	<0.001	<0.001	<0.001	<0.001	<0.001
P	3	<0.001	<0.001	<0.001	<0.001	<0.001
RA × P	9	<0.001	0.013	<0.001	<0.001	<0.001

Means followed by different letters under the same factor are significantly different according to Tukey’s HSD test (p ≤ 0.05).

**TABLE 6:** Impact of root activator (RA) and phosphorus (P) levels on P in seeds (%), P in straw (%), total P uptake (kg ha*^–^*^1^), P recovery efficiency (PRE), and P use efficiency (PUE).

Factor		P in seed	P in straw	total P uptake	PRE	PUE
**RA**						
RA0 (control)		0.25 ± 0.021^d^	0.09 ± 0.004^d^	3.45 ± 0.34^d^	5.36 ± 0.86^d^	12.78 ± 0.98^d^
RA1 (1.25 l ha^–1^)		0.29 ± 0.018^c^	0.10 ± 0.005^c^	6.20 ± 0.73^c^	9.57 ± 0.80^c^	18.06 ± 1.17^c^
RA2 (2.50 l ha^–1^)		0.48 ± 0.017^b^	0.12 ± 0.004^b^	10.22 ± 0.68^b^	15.97 ± 1.03^b^	21.15 ± 1.38^b^
RA3 (3.75 l ha^–1^)		0.55 ± 0.015^a^	0.22 ± 0.016^a^	14.34 ± 0.83^a^	22.01 ± 1.15^a^	22.17 ± 1.46^a^
**P**						
P0 (control)		0.31 ± 0.040^d^	0.10 ± 0.09^c^	5.42 ± 0.93^d^		
P1 (50 kg P_2_O_5_ ha^–1^)		0.37 ± 0.046^c^	0.12 ± 0.010^b^	7.54 ± 1.23**^c^**	15.09 ± 1.22^a^	22.92 ± 2.17^a^
P2 (75 kg P_2_O_5_ ha^–1^)		0.43 ± 0.036^b^	0.15 ± 0.013^a^	10.00 ± 1.31^b^	13.33 ± 1.25^b^	18.15 ± 1.88^b^
P3 (100 kg P_2_O_5_ ha^–1^)		0.47 ± 0.031^a^	0.16 ± 0.012^a^	11.26 ± 1.35^a^	11.26 ± 0.97^c^	14.56 ± 1.46^c^
**Interaction**						
RA0	P0	0.17 ± 0.013^j^	0.07 ± 0.002^k^	1.95 ± 0.10^i^		
	P1	0.20 ± 0.013^i,j^	0.08 ± 0.002^j,k^	2.83 ± 0.14^h^	5.67 ± 0.29^g^	16.47 ± 0.07^f^
	P2	0.29 ± 0.010^h^	0.10 ± 0.001^i^	4.10 ± 0.016^g^	5.55 ± 0.20^g^	12.00 ± 0.15^h^
	P3	0.34 ± 0.015^g^	0.10 ± 0.001^i^	4.87 ± 0.20^g^	4.87 ± 0.18^g^	9.85 ± 0.09^i^
RA1	P0	0.19 ± 0.014^j^	0.08 ± 0.003^k^	3.26 ± 0.17^h^		
	P1	0.23 ± 0.017^i^	0.09 ± 0.003^j^	4.67 ± 0.25^g^	9.34 ± 0.32^f^	22.57 ± 0.10^c^
	P2	0.34 ± 0.010^g^	0.11 ± 0.001^h^	7.44 ± 0.08^f^	9.92 ± 0.19^f^	16.83 ± 0.11^e,f^
	P3	0.42 ± 0.016^f^	0.12 ± 0.001^g,h^	9.44 ± 0.33^e^	9.44 ± 0.28^f^	14.79 ± 0.23^g^
RA2	P0	0.39 ± 0.010^f^	0.10 ± 0.002^i^	6.71 ± 0.11^f^		
	P1	0.48 ± 0.012^d,e^	0.12 ± 0.002^f,g^	9.79 ± 0.16^e^	19.58 ± 0.35^c^	25.98 ± 0.14^b^
	P2	0.52 ± 0.012^c,d^	0.13 ± 0.001^e,f^	11.78 ± 0.15^d^	15.71 ± 0.26^d^	21.04 ± 0.10^d^
	P3	0.53 ± 0.013^c^	0.13 ± 0.003^e^	12.61 ± 0.20^c,d^	12.61 ± 0.22^e^	16.44 ± 0.09^f^
RA3	P0	0.48 ± 0.018^e^	0.16 ± 0.003^d^	9.74 ± 0.19^e^		
	P1	0.55 ± 0.019^b,c^	0.19 ± 0.003^c^	12.88 ± 1.18^c^	25.77 ± 0.21^a^	26.65 ± 0.20^a^
	P2	0.57 ± 0.013^a,b^	0.25 ± 0.004^b^	16.61 ± 0.23^b^	22.15 ± 0.37^b^	22.71 ± 0.17^c^
	P3	0.60 ± 0.012^a^	0.27 ± 0.003^a^	18.11 ± 0.26^a^	18.11 ± 0.28^c^	17.14 ± 0.11^e^

**ANOVA**	**Df**	***P-value***				

RA	3	<0.001	<0.001	<0.001	<0.001	<0.001
P	3	<0.001	<0.001	<0.001	<0.001	<0.001
RA × P	9	<0.001	<0.001	<0.001	<0.001	<0.001

Means followed by different letters under the same factor are significantly different according to Tukey’s HSD test (p ≤ 0.05).

The seeds were inoculated with the proper strain of *Rhizobium* (*Rhizobium leguminosarum*). All other agricultural practices, comprising drip irrigation to the reference crop evapotranspiration (ET0), weed, disease, and pest control, were performed according to the recommendations of the commercial production of lentil.

### Measurements of root and physiological parameters

After 55 DAS, five plants from each plot were collected randomly to determine root traits. Plants were gently uprooted and washed with tap water to remove the soil from the roots. Then roots were washed with distilled water and blotted with tissue paper. The root length (cm) was measured from the collar region to the tip of the main root, and the number of total nodules was counted on the main and lateral roots. Nodules were detached from the roots and cut into two pieces, and observed for the inside color. Pink/red nodules were recorded as healthy and active in N_2_ fixation. The remaining nodules with other colors were classified as inactive to fix N_2_. The active nodules and total root were dried in the oven at 70°C for 48 h and then weighed. Nitrogenase enzyme activity (μmol C_2_H_4_ g^–1^ nodule DW h^–1^) was estimated using an acetylene reduction assay as described by Hardy et al. (1973). At 55 DAS, chlorophyll *a*, chlorophyll *b*, and carotenoids (mg g^–1^ FW) were determined according to Hiscox and Israelstam (1979).

### Measurements of seed yield and its attributes

At maturity (130 DAS), ten plants were taken randomly from the inner rows of each plot to measure plant height (cm), number of branches plant^–1^, and number of pods plant^–1^. All plants in each plot were harvested to determine the 1,000-seed weight (g), seed yield (kg ha^–1^), and biological yield (kg ha^–1^).

### Measurements of nitrogen and phosphorus

#### Measurements of nitrogen

The total N in seed and straw was determined using the Micro-Kjeldahl method as described by Horwitz et al. (2000). N in seed and straw, N uptake, protein percentage, N recovery efficiency (NRE), and N use efficiency (NUE) were calculated according to the following equations.

N uptake (kg ha^–1^) = N in seed (kg ha^–1^) + N in straw (kg ha^–1^)
N in seeds (kg ha^–1^) = Seed N % × seed yield (kg ha^–1^)/100
N in straw (kg ha^–1^) = Straw N % × straw yield (kg ha^–1^)/100
Protein % in seeds was calculated by multiplying N% by a factor of 6.25
NRE = Total N uptake (kg ha^–1^) × 100/N applied (kg ha^–1^)
NUE = Seed yield (kg ha^–1^)/N applied (kg ha^–1^)

#### Determination of phosphorus

In an acid mixture of HNO_3_ and HClO_4_, samples of seeds and straw were digested. Then, P was determined by developing color by color reagent (ammonium molybdate, ammonium vanadate, and nitric acid) with a spectrophotometer ANA-730 at 470 nm wavelength after calibrating with P standards (Horwitz et al., 2000). The accumulated total P in seeds and straw were used to calculate P recovery efficiency (PRE), and P use efficiency (PUE), according to the following equations:

PRE = Total P uptake (kg ha^–1^) × 100/applied P (kg ha^–1^)
PUE = Seed yield (kg ha^–1^)/applied P (kg ha^–1^)

### Statistical analysis

The obtained data were subjected to normality distribution of the residuals and homogeneity of variances prior to analysis of variance (ANOVA) using Shapiro–Wilk and Bartlett’s tests (Bartlett, 1937; Shapiro and Wilk, 1965). The combined data of two seasons were subjected to ANOVA using R statistical software version 4.4.1. Differences among the treatments were separated by Tukey’s HSD test (*P* ≤ 0.05).

Regression analysis was performed between total N and P uptake as dependent variables and root traits as independent variables. A biplot of principal component analysis (PCA) was performed to study the relationship among the studied traits.

## Results

### Root traits

The applied RA, P fertilization, and their interaction displayed significant impacts on the measured root traits; thus, significantly enhanced the root length, number of active nodules plant^–1^, dry weight of active nodules, total root dry weight, and nitrogenase enzyme activity (Table 1). The application of RA at 3.75 l ha^–1^ (RA3) and P level of 100 kg P_2_O_5_ ha^–1^ (P3) exhibited the highest values of all root traits. Compared to untreated control (RA0), the RA3 treatment enhanced the root length by 88.5%, the total number of nodules plant^–1^ by 128.6%, and number of active nodules plant^–1^ by 133.0%. Similarly, the dry weight of active nodules, the dry weight of total root, and nitrogenase activity increased by 131.6, 45.8, and 95.8%, respectively, when RA3 was applied (Table 1).

The P3 treatment, which is the highest P treatment in this study, also enhanced all the root traits tested. For example, the root length, total number of nodules plant^–1^, number of active nodules plant^–1^, dry weight of active nodules, total root dry weight, and nitrogenase activity increased by 32.8, 66.4, 66.0, 69.6, 13.8, and 46.7%, respectively, compared to P0 treatment (control) (Table 1). The combination of RA3-P2 and RA3-P3 exhibited the highest enhancement in the root length by 142.0 and 144.2%, the total number of nodules plant^–1^ by 257.1 and 277.0%, number of active nodules plant^–1^ by 247.1 and 257.4%, respectively, compared to the corresponding control treatment (RA0-P0) (Table 1). In addition, the active nodules dry weight increased by 242.5 and 257.5%, total root dry weight increased by 56.5% and 62.4% and the nitrogenase activity increased by 147.5 and 148.1%, respectively (Table 1).

### Physiological parameters

The treatments of RA and P fertilization significantly affected chlorophyll *a*, *b*, and carotenoids contents (Table 2). Increasing the RA rate to 3.75 l ha^–1^ (RA3) caused considerable increases in chlorophyll *a*, *b*, and carotenoids by 75.0, 112.0, and 274.2%, respectively, compared to untreated control (RA0). The highest P level exhibited the highest contents of these photosynthetic pigments (Table 2).

For example, the P3 increased the contents of chlorophyll *a*, *b*, and carotenoids in lentil plants by 35.0, 48.4, and 89.6% compared to control plants without any P fertilizer applied (P0). The interactive effect of RA3-P2 and RA3-P3 showed the highest photosynthetic pigments with an increase of 120.9 and 139.5% in chlorophyll *a*, 173.7 and 226.3% in chlorophyll *b*, and 605.6 and 733.3% in carotenoids, respectively, compared to non-treated control (RA0-P0) (Table 2).

### Yield and its attributes

Treatments with either RA or P fertilization significantly affected yield and its attributes in plants (Table 3). The increasing rate of RA to 3.75 l ha^–1^ (RA3) enhanced plant height by 71.5%, number of branches plant^–1^ by 23.1%, number of pods plant^–1^ by 66.2%, 1,000-seed weight by 29.1%, seed yield by 77.4%, and biological yield by 84.5%, compared to those in the RA0 control (Table 3). Likewise, plants treated with the highest P level showed the highest values of yield traits. P3 treatment enhanced plant height by 20.5%, number of branches plant^–1^ by 50.3%, number of pods plant^–1^ by 36.8%, 1,000-seed weight by 19.1%, seed yield by 52.9%, and biological yield by 32.0%, compared to plants without P application (P0) (Table 3).

The combinations of RA and P fertilization displayed significant effects on yield traits. The application of RA3-P3 and RA3-P2 on plants exhibited the highest values for all evaluated agronomic traits (Table 3). We noticed that these interactions, RA3-P2 and RA3-P3, resulted in an enhancement in plant height by 106.4 and 107.7%, number of branches plant^–1^ by 56.3 and 83.3%, number of pods plant^–1^ by 125.4 and 128.4%, 1,000-seed weight by 48.0 and 47.6%, seed yield by 169.5 and 171.2%, and biological yield by 131.4 and 139.7%, respectively, compared to non-treated control RA0-P0 (Table 3).

### Quality traits

Seed protein yield (SPY) and seed carbohydrate yield (SCY) of lentil plants were significantly affected by RA, P fertilization, and their interaction (Table 4). Thus, the highest values of SPY and SCY in plants treated with RA were recorded by RA3 displaying 163.8% and 92.3%, respectively, compared to those in RA0 (Table 4). Among all P treatments, plants treated with P3 showed the highest values of SPY and SCY by 87.2% and 66.7%, respectively, compared to plants treated with no P fertilizer (P0) (Table 4).

The interaction between RA3 with P2 and RA3 with P3 achieved the highest values of SPY, surpassing the untreated control (RA0-P0) by 382.6 and 389.4%, respectively. SCY was also increased by 216.5 and 218.1%, respectively, compared to RA0-P0 (Table 4).

### Nitrogen parameters

All studied N parameters were significantly affected by the RA, P fertilization, and their interaction. The parameters were increased as the rates of the RA or levels of P increased; thus, the highest measurements were recorded at 3.75 l ha^–1^ (RA3) or 100 kg P_2_O_5_ ha^–1^ (P3), compared to RA0 or P0 control treatments (Table 5). Our results displayed considerable enhancement of N% in seed, N% in straw, total N uptake, NRE, and NUE values in plants treated with the highest rate of RA (RA3) by 50.2, 42.4, 164.8, 164.7, and 77.3%, respectively, compared to the untreated control (RA0) (Table 5).

Lentil plants supplemented with the highest P level (P3) boosted the N% in seed by 24.5%, N% in straw by 21.6%, total N uptake by 78.8%, NRE by 78.7%, and NUE by 52.8% as compared to non-added P control (P0) in the same order (Table 5). Moreover, the highest amounts of N% in seeds were found in RA3-P2 and RA3-P3 treatments, exhibiting a 79.1% and 80.4% increase, respectively, when compared to the untreated control (RA0-P0) (Table 5). Likewise, treatments of RA3-P2 and RA3-P3 enhanced N% in straw by 56.7 and 70.0%, respectively, compared to the untreated control. Total N uptake, NRE, and NUE values exceeded the values of the untreated control (RA0-P0) by 351.0, 350.7, and 169.6%, respectively, in response to RA3-P2 treatment, and 366.4, 366.3, and 171.4%, respectively, in response to RA3-P3 treatment (Table 5).

### Phosphorus parameters

The P contents in seed and straw were also estimated to evaluate P physiological parameters affected by the application of the RA and/or P fertilization. The results revealed the application of RA3 enhanced P% in seed, P% in straw, total P uptake, PRE, and PUE values in lentil plants by 120.0, 144.4, 315.7, 310.6, and 73.5%, respectively, compared to RA0 control plants (Table 6). When plants were supplied with P3, there were significant increases in P% in seed, P% in straw, and total P uptake by 51.6, 60.5, and 107.8%, respectively, compared to those of P0 treatment (Table 6).

Thus, the interactive treatment between RA and P fertilization significantly impacted P parameters. The combined RA3-P3 enhanced P% in seeds, P% in straw, and total P uptake in plants by 252.9, 285.7, and 428.7%, respectively, in comparison to those plants that received no treatment (RA0-P0) (Table 6). Similarly, RA3-P2 boosted P% in seeds, P% in straw, and total P uptake in plants by 235.3, 257.1, and 751.3%, respectively, compared to untreated control (Table 6). However, the highest PRE and PUE values were obtained in RA3-P1, followed by RA3-P2 and RA3-P3 combined treatment (Table 6).

### Regression analysis

**FIGURE 1:**
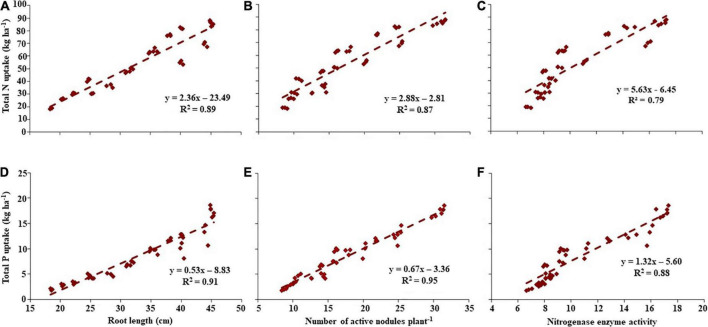
Regression relationship between total nitrogen (N) or phosphorus (P) uptake and root traits. Regression relationship of total **(A–C)** N and **(D–F)** P uptake on **(A,D)** root length; **(B,E)** number of active nodules plant*^–^*^1^; and **(C,F)** nitrogenase enzyme activity.

The regression analysis exhibited a positive linear relationship between the total N uptake with the root length, number of active nodules plant^–1^, and the nitrogenase enzyme activity in lentil (Figures 1A–C). From *R*^2^ values, it was observed that the three root traits were highly associated with total N uptake. Likewise, the three root parameters displayed a positive linear relationship with total P uptake (Figures 1D–F).

### Interrelationship among the assessed treatments and traits

**FIGURE 2:**
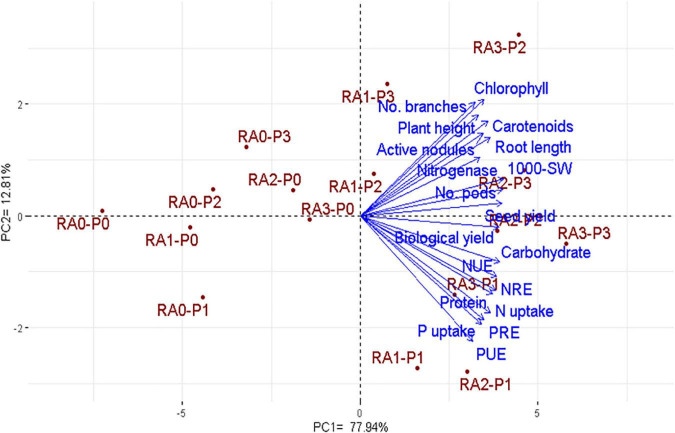
Principal component analysis (PCA) biplot for the assessed treatments of RA and P applications and the evaluated traits of lentil over the two growing seasons. RA, root activator; P, phosphorus; NUE, N use efficiency; NRE, N recovery efficiency; PUE, P use efficiency; PRE, P recovery efficiency.

PCA was employed to study the relationship among the assessed treatments and traits, as displayed in Figure 2. The first two PCAs exhibited 90.75% of the variability. The PCA1 accounted for 77.94% of the variation and was associated with the level of assessed treatments of RA and P application from the untreated control (RA0-P0) on the extreme left to the highest level on the extreme right (RA3-P3) (Figure 2).

The highest two levels of both treatments of RA (RA2 and RA3) and P fertilization (P2 and P3) had slight multi-dimensional space as exhibited by the small distances of plots along PCA1, compared to the corresponding controls (RA0 and P0) and the low RA1 and P1 levels which were spread apart and with more dissimilarity (Figure 2). The evaluated root, physiological, agronomic, and quality traits were positively associated with the high levels of RA (RA2 and RA3) and P fertilization (P2 and P3) on the PCA1, which is consistent with the obtained results in Tables 1–6. Thereupon, the PCA biplot is emphasizing the foregoing displayed results (Figure 2).

## Discussion

Newly reclaimed sandy soils suffer from nutrient deficiency and various environmental stresses (Mansour et al., 2021). Accordingly, it is imperative to find ecofriendly approaches to enhance plant growth and production under these poor conditions (Desoky et al., 2021b). The present study was performed in newly reclaimed low-fertility sandy soil containing a very low concentration of nutrients, particularly P and N, compared to the other normal fertile soils. Hence, the impact of RA and P application in different levels were assessed on lentil morphological, physiological, agronomic, and quality characteristics.

The recommended P rate in the newly reclaimed low-fertility sandy soil was 75 kg P_2_O_5_ ha^–1^; in addition to the lower (50 kg P_2_O_5_ ha^–1^) and higher (100 kg P_2_O_5_ ha^–1^) treatment rates were applied. The obtained results revealed that the three P levels significantly boosted the root traits (root length, number of active nodules, dry weight of active nodules, total root dry weight, and nitrogenase activity) with the maximum values from the highest P level. Roots can control water and nutrient uptake and provide anchoring and mechanical support. P is a critical element for stimulating root development and growth (Singh and Singh, 2016; Du et al., 2022), and plays a crucial role in cell division, metabolic activities, nucleus formation, nodulation, N_2_ fixation, as well as starch utilization (Singh et al., 2005; Singh and Singh, 2016). The applied P, particularly the highest level (P3), considerably enhanced all root traits and exhibited strong root growth. Vigorous root system with high root length, active nodules, and dry weight ensured better efficiency in uptaking macro and micronutrients (Wang et al., 2016; Su et al., 2022).

Furthermore, P3 enhanced N_2_ fixation efficiency and N uptake by increasing the number of active nodules, nitrogenase activity, and dry weight of active nodules. The relative superiority of root traits associated with P3 treatment was in agreement with previous results. In this respect, Singh et al. (2005) have demonstrated that each increment in P fertilization can enhance the main root length and dry weight. P application enhanced primary root length, total surface area, total root tips, root forks, total dry weight, and root dry weight in lentil plants (Ramtekey et al., 2021). Similarly, the increase in P levels stimulated the N_2_ fixation efficiency and N uptake in lentils by improving active nodules and their dry weight plant^–1^ (Jindal et al., 2008; Rasheed et al., 2010). Mohamed et al. (2021) have also disclosed that increasing the P level boosted the root dry weight and absorption of macronutrients in common bean.

The RA application substantially stimulated all root traits with relative superiority to RA3. One strategy to enhance crop acquisition efficiency of low-available mineral elements, such as N, P, K, Fe, Mn, Cu, Zn, and Mo, in sandy soil is to improve root traits (Gahoonia and Nielsen, 2004; Gahoonia et al., 2006). In the current study, we applied the RA, which contained IBA, GA_3_, NAA, and K humate. Previously, the application of the plant growth promoters IBA, GA_3_, and NAA enhanced root growth, expansion of root hairs, and cotyledon cells (Chhun et al., 2004; Elmongy et al., 2018). The plant growth regulators have been reported to inhibit primary root elongation but stimulate lateral prolific root hair formation (Pulok et al., 2015). Besides, K humate has a positive impact on the root growth and a number of nodules; thus, this effectively enhances N_2_ fixation and nutrient uptake (Rafique et al., 2021). Improving root traits attributed to the application of plant growth promoters has been reported extensively in other studies (Chhun et al., 2004; Pulok et al., 2015; Elmongy et al., 2018; Mao et al., 2018).

The plant pigments, including chlorophyll *a*, *b*, and carotenoids, are indispensable in the photosynthesis process, which provide the essential requirements for plant development (Desoky et al., 2021d; Khan et al., 2021). The RA and P applications significantly enhanced chlorophyll and carotenoids content compared to untreated control. The positive impact of RA on the contents of chlorophyll *a*, *b*, and carotenoids could be attributed to the applied plant regulators IBA, GA_3_, and NAA (Piotrowska-Niczyporuk et al., 2012). The plant growth regulators improve the source-sink relationship through stimulating transportation and distribution of accumulates, enhancing photosynthesis and sink formation, and invigorating the photo-assimilates translocation (Li et al., 2016; Mustafa et al., 2016; Desoky et al., 2021a; Rafique et al., 2021). Likewise, the P application induced higher contents of chlorophyll *a*, *b* and carotenoids compared to the non-added control. P is an imperative nutritional component, and its lack markedly influences plant pigments (Frydenvang et al., 2015; Carstensen et al., 2018). The high level of both treatments (RA3 and P3) exhibited the maximum values of photosynthesis pigments.

Plant growth and productivity are resulted from the integration of different metabolic and physiological responses. RA and P applications exhibited positive impacts on the root and physiological parameters. Both treatments increased the root length, number of active nodules, and nitrogenase activity, which enhanced N*_2_* fixation and total N uptake. Likewise, RA and P applications increased the contents of chlorophyll *a*, *b*, and carotenoids. In particular, the highest levels of RA and P application displayed the maximum enhancement in root traits and physiological parameters; thus, this was reflected in boosting nutrient absorption and plant growth. Correspondingly, the high level of RA (3.75 l ha^–1^) and P fertilization (100 kg P_2_O_5_ ha^–1^) displayed the uppermost plant height, number of branches plant^–1^, number of pods plant^–1^, 1,000-seed weight, seed yield, and biological yield.

Similarly, the recommended P level (75 kg P_2_O_5_ ha^–1^, P2) and the high level of RA (3.75 l ha^–1^, RA3) displayed non-significant differences in plant height, number of branches plant^–1^, 1,000-seed weight, and seed yield, compared to the high P level (100 kg P_2_O_5_ ha^–1^, P3). For economic and environmental purposes, it is highly recommended to use the coapplication of P2 and RA3. In this context, several studies have reported substantial improvement in seed yield and its attributes by applying plant growth promoters (Balyan and Singh, 2005; Singh et al., 2005; Togay et al., 2008; Rafique et al., 2021). Similarly, the role of P application in enhancing seed yield and its components has been previously elucidated (Singh et al., 2005; Togay et al., 2008; Nget et al., 2022; Yang et al., 2022).

Increasing N and protein content in lentil seeds was emphatically associated with applying RA and phosphorous fertilization compared to untreated control. The highest levels of RA3 and P3 displayed the uppermost values of quality parameters. Expanding protein concentration in seeds increases the nutritional value of lentil seeds. The protein increment in lentil seeds could be resulted from promoting N*_2_* fixation, biological activities, and physiological capabilities (Togay et al., 2008; Rasheed et al., 2010; Sital et al., 2011).

## Conclusion

The current research clarified the potential morphological, physiological, agronomic, and quality parameters of lentil plants to different levels of RA and P fertilization. RA and P applications enhanced lentil growth and productivity compared to untreated control by invigorating root traits, nodulation, and physiological parameters. The highest yield traits (plant height, 1,000-seed weight, seed yield ha^–1^) and quality characters (protein and carbohydrate) were achieved by the coapplication of RA and P fertilization at 3.75 l ha^–1^ and 75 kg P_2_O_5_ ha^–1^ with no significant differences with 100 kg P_2_O_5_ ha^–1^. Subsequently, it is recommended to apply RA and P fertilization to lentil plants at the aforementioned rates to enhance plant growth, yield, and quality and improve agricultural and environmental sustainability under newly reclaimed low-fertility soil.

## Data availability statement

The original contributions presented in this study are included in the article/Supplementary material, further inquiries can be directed to the corresponding author/s.

## Author contributions

MAE-h, YA-E, MR, KE-T, SA, and ME-t conceived and designed the experiments. EM, KE-T, and SA analyzed the data and drafted the manuscript. MAE-h, EM, KE-T, SA, and ME-t wrote and edited the final manuscript. All authors read and approved the final version of the manuscript.
