# Supplementary material for: Impact on plant productivity under low-fertility sandy soil in arid environment by revitalization of lentil roots
Source: Front Plant Sci. 2022 Aug 4;13:937073. doi: 10.3389/fpls.2022.937073 (PMC9386484; doi:10.3389/fpls.2022.937073)
Supplement: Supplementary file 1 [file Data_Sheet_1.pdf]

Supplementary material

Impact on plant productivity under low fertility sandy soil in arid environment by revitalization of lentil roots

Mohamed A. Abd El-hady[1], Yasser M. Abd-Elkrem[1], Mohamed O. A. Rady[2], Elsayed Mansour[3], Khaled A. El-Tarabily[4,5,6]*, Synan F. AbuQamar[4,*] and Mohamed E. El-temsah[1]

*** Correspondence:**
Khaled El-Tarabily: ktarabily@uaeu.ac.ae
Synan AbuQamar: sabuqamar@uaeu.ac.ae

Supplementary material

Supplementary Table S1. Some physio-chemical properties of the experimental soil (average over both seasons).

Supplementary Table S2. Monthly average minimum temperature (Tmin, °C), maximum temperature (Tmax, °C), precipitation (Prec) and relative humidity (RH) in the two growing seasons.

Supplementary Table S1. Some physio-chemical properties of the experimental soil (average over both seasons).

Physical analysis			
Soil texture	Clay (%)	Silt (%)	Sand (%)
Sandy soil	6.3	1.6	92.1

Chemical analysis									
EC $dS.m^{-1}$	pH	Soluble cations (Milliequivalents l^{-1})				Soluble anions (Milliequivalents l^{-1})			Organic matter (%)
		K^+	Na^+	Ca^{+2}	Mg^{+2}	Cl^-	HCO_3^-	SO_4^{-2}	
0.88	7.50	0.38	4.18	2.05	2.63	4.64	3.74	0.81	0.41

Supplementary Table S2. Monthly average minimum temperature (Tmin, °C), maximum temperature (Tmax, °C), precipitation (Prec) and relative humidity (RH) in the two growing seasons.

Month	Tmin (°C)	Tmax (°C)	Prec (mm)	RH (%)
			**2018–2019**	
November	13.43	25.53	0.21	61.42
December	9.07	19.58	0.37	67.13
January	5.41	17.80	0.05	54.83
February	6.53	19.96	0.14	56.24
March	8.46	23.11	0.24	56.34
April	11.72	27.49	0.07	48.64
			**2019–2020**	
November	13.96	27.48	0.00	57.75
December	8.93	20.32	0.84	66.94
January	6.65	17.53	0.45	69.76
February	7.36	19.77	1.19	68.53
March	9.33	24.13	1.54	60.53
April	11.57	26.64	1.87	57.72
